# Rediscovery of *Craspedosorus* and Plastome‐Based Evidence for Its Synonymy With *Leptogramma* (Thelypteridaceae)

**DOI:** 10.1002/ece3.73498

**Published:** 2026-04-22

**Authors:** Jing Zhao, Jian‐Jun Yang, Zhong‐Yang Li, Chuang‐Jie Huang, Ting Chen, Yong‐Li Zhao, Zhao‐Rong He, Chao‐Shan Gu, Xin‐Mao Zhou

**Affiliations:** ^1^ School of Ecology and Environmental Science Yunnan University Kunming Yunnan China; ^2^ College of Life Sciences Gannan Normal University Ganzhou Jiangxi China; ^3^ Administration Bureau of Yunnan Wumengshan National Nature Reserve Zhaotong Yunnan China; ^4^ School of Life Sciences Yunnan University Kunming Yunnan China

**Keywords:** *Leptogramma*, rare species, rediscovery, *Stegnogramma*, Thelypteridaceae

## Abstract

Yunnan Province is the richest region in China for lycophytes and ferns. Its unique geological history and climatic conditions have nurtured numerous endemic and rare plant species. *Craspedosorus*, a monotypic genus of Thelypteridaceae containing only *Craspedosorus sinensis*, is a genus endemic to Yunnan, China. It was first discovered and described in the 1970s. However, since its initial discovery in 1973, there have been no subsequent field observations or collection records. Moreover, due to a lack of molecular data, *Craspedosorus* remains the only fern genus in China without such data and has long had a controversial systematic position. This study rediscovered a population of only 15 mature individuals in Yongshan County, Zhaotong City, Yunnan Province, 52 years after its initial discovery. Using flow cytometry, it was confirmed that *Craspedosorus sinensis* is diploid, with a genome size of approximately 5.12 Gb. Molecular phylogenetic analyses further determined that *C. sinensis* belongs to Section *Haplogramma* (K. Iwatsuki) L.Y. Kuo & Y.H. Chang of the genus *Leptogramma,* and *Craspedosorus* therefore should be treated as a synonym of *Leptogramma*. This research is significant for understanding the taxonomy, distribution, and phylogeny of *Leptogramma*.

## Graphical abstract

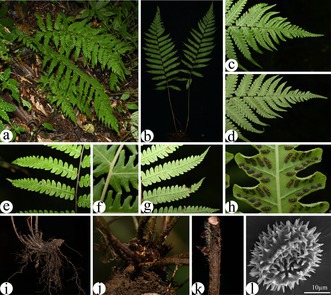

## 1 Introduction

**FIGURE 1:**
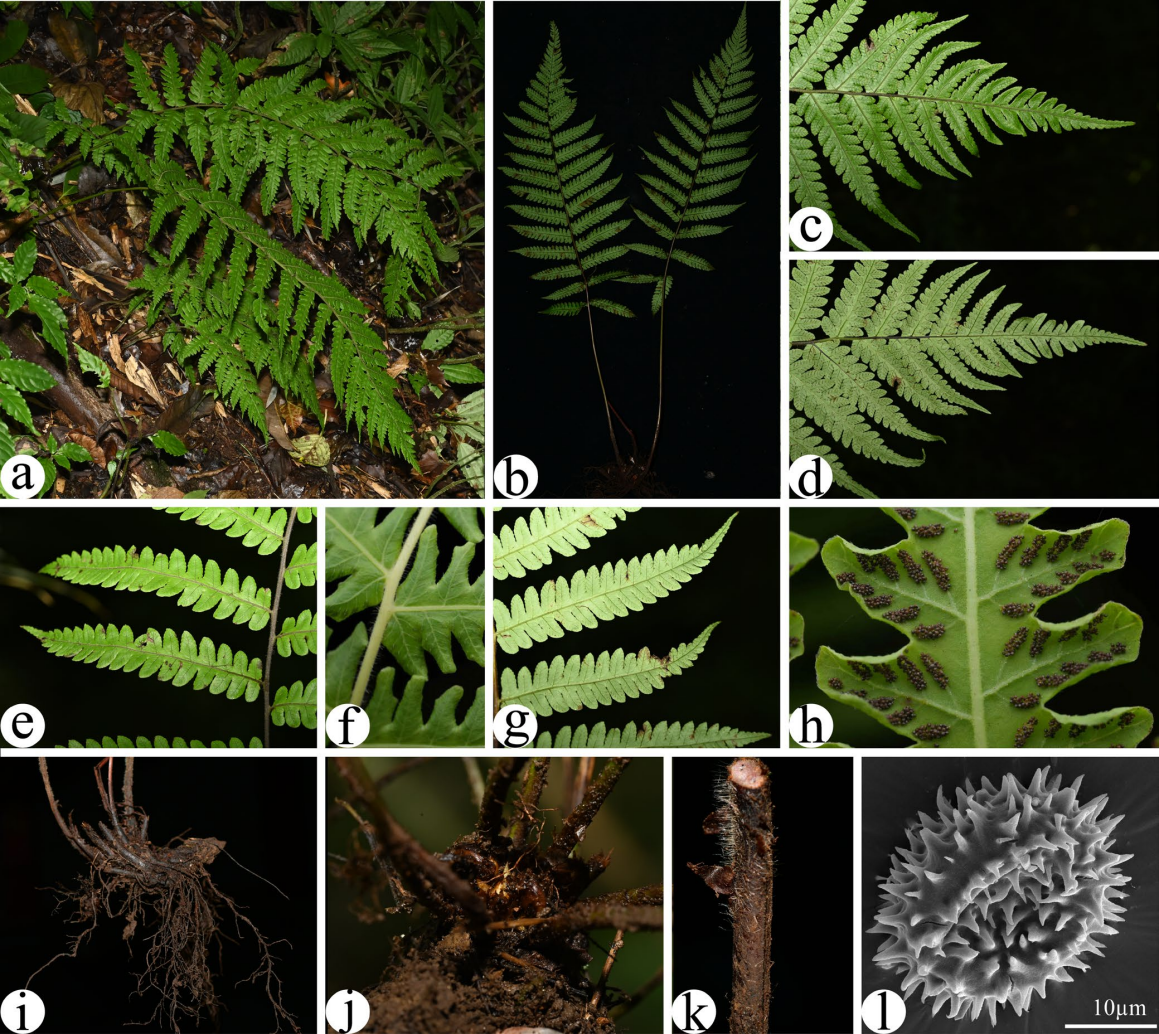
Habitat and morphology of *Craspedosorus sinensis*. (a) Habitat. (b) Habit. (c) Dorsal view of portion of fertile frond. (d) Ventral view portion of fertile frond. (e) dorsal view of laminae. (f) Short rough hairs on rachises. (g) Ventral view of laminae. (h) Sporangia. (i, j) Rhizomes. (k) Stipe. (l) Spore. Photos taken by Chuan‐Jie Huang (a–e, g, i–k), Zhong‐Yang Li (f, h), and Dan‐Ni Ma (l).

The Chinese endemic monotypic genus *Craspedosorus* Ching et W.M. Chu was first described by Prof. R.C. Ching (Ching 1978) based on *C. sinensis* Ching et W.M. Chu (voucher: *W.M. Chu 4938*; Figure 1). *C. sinensis* was firstly collected by Prof. W.M. Chu in 1973 in Suijiang County, Yunnan Province, China, at an elevation of approximately 1500 m. However, whether *Craspedosorus* should be considered an independent genus or a synonym of *Stegnogramma* sensu lato (*s.l*.) has remained controversial since its establishment. *Craspedosorus* has been recognized by the Flora of China (FOC; Lin et al. 2013) and Flora Reipublicae Popularis Sinicae (FRPS; Shing 1999) as closely related to *Leptogramma*. However, A.R. Smith, a co‐author of the FOC, argued that the diagnostic characters of *Craspedosorus* are insufficient to distinguish it from *Leptogramma*. Zhang (2012) proposed that *Craspedosorus* should be classified as a member of *Stegnogramma* Blume *s.l*. based on morphological data. Kuo et al. (2020) divided *Stegnogramma s.l*. into two genera: *Stegnogramma* sensu strict (*s.s*.) and *Leptogramma*, with the latter including two sections (*L*. sect. *Leptogramma* and *L*. sect. *Haplogramma*), instead of three genera (Ching 1963) or one genus with four sections as suggested by Iwatsuki (1963). In their study, *C. sinensis* was transferred into *Leptogramma*, and based on geographical distribution, the presence of multicellular hairs on stipes, and other morphological evidence, *C. sinensis* was further assigned to *L*. sect. *Haplogramma*. Fawcett and Smith (2021) also advocated merging *Craspedosorus* into *Leptogramma* J. Sm. based on morphological evidence. Morphologically, *C. sinensis* is mostly similar to species of *Leptogramma* J. Sm in laminar outline, segment shape, venation, and sori (He and Zhang 2012; Kuo et al. 2020). However, it differs from *Leptogramma* by its taller habit (up to 1.1 m), more pinnae (ca. 25 pairs), and pinnae that are separated from the rachis except at the apex (Ching 1978; Chu 2006).

The difficulty of using micromorphological features to distinguish genera within Thelypteridaceae is evident, and molecular evidence offers a more objective basis for the treatment of genera, especially for morphologically diverse taxa (Ching 1936, 1940; Morton 1963; Pichi Sermolli 1970; Smith and Cranfill 2002; Almeida et al. 2016; PPG I 2016; Patel et al. 2019; Liu et al. 2020; Fawcett and Smith 2021; Fawcett et al. 2021; Zhou and He 2025). The phylogenetic position of *Craspedosorus* remains controversial, and substantial evidence to clarify its placement is still lacking. The primary reason for the uncertain phylogenetic position of *C. sinensis* is the extreme rarity of this species. Since the initial discovery of *Craspedosorus*, there have been no further records of *C. sinensis* in the field, including at the type locality. China's vast land area encompasses diverse vegetation types, allowing ferns to adapt widely across various flora throughout the country (Qian et al. 2021). Over the past 20 years, research advancements have led to changes in the number of fern families, genera, and species distributed in China. According to FOC (38 families, 177 genera, approximately 2219 species) (Wu et al. 2013), FRPS (61 families, 231 genera, approximately 2600 species) (Wu and Chen 2004), and the Catalogue of Life China (42 families, 191 genera, approximately 2693 species) (Liu et al. 2025), a combined total of 68 families and 280 genera have been recorded in China. Among the pteridophyte genera recorded in China, only one genus, *Craspedosorus*, lacks molecular data. The addition of molecular data from *Craspedosorus* will complete the final piece of the puzzle regarding pteridophyte genetic diversity at the genus level and will enhance the study of pteridophyte phylogeny in China.

In this study, we successfully obtained the plastome of *Craspedosorus sinensis* using next‐generation sequencing. Simultaneously, we carefully examined the morphological characteristics of the type specimens and collected detailed morphological data. Our objectives are to (1) explore the phylogenetic position of *Craspedosorus*; (2) compare the morphological differences between *C. sinensis* and closely related species based on character state reconstruction results; and (3) infer the evolution of major anatomical, macromorphological, and spore features in *Craspedosorus*.

## 2 Materials and Methods

### 2.1 Investigation and Morphological Observations

Two extensive field investigations were conducted in 2024 and 2025 in both type locality and adjacent areas. *Craspedosorus sinensis* was rediscovered (elev. 1784 m, 28.243 N, 103.948 E) in Xisha Town, Yongshan County, Yunnan Province, China. Voucher specimens were deposited at PYU (herbarium acronyms follow Index Herbariorum by Thiers 2024). Macromorphological data were obtained from field observations, herbarium specimens, and literature (e.g., Ching 1978; Chu 2006). Gross morphology was examined, and photographs were taken using an SMZ1270 stereo microscope (Nikon, Japan). Spores of the species were coated with gold using the BAL‐TEC SCD 005 Cool Sputter Coater (BAL‐TEC AG., Liechtenstein) and imaged with a QUANTA 200 Scanning Electron Microscope (SEM; FEI Co., USA) at 25 kV at Yunnan University, Kunming, China.

### 2.2 Genome Size Assessment

Fresh and healthy leaves (ca. 5 g) were collected from living plants introduced from the field and frozen at −80°C. Flow cytometry was performed using a BD FACScalibur. Tender leaves of *Pisum sativum* L. (genome size = 3.8 Gb) one month after germination served as the reference. Genome sizes were determined based on the sample/standard ratio.

### 2.3 DNA Extraction and Sequencing

Total genomic DNA was extracted from silica‐dried material using the TIANGEN plant genomic DNA extraction kit (TIANGEN Biotech., Beijing, China) following the manufacturer's protocols. The DNA samples were sent to Biomaker Technology Co. Ltd. (Beijing, China) for library construction and next‐generation sequencing. A paired‐end library with an insert size of 350 bp was constructed, and sequencing was performed using the Illumina Nova 6000 platform. The raw Illumina data generated three gigabases (3 Gb). The raw reads were subsequently trimmed for quality using Fastp v0.23.1 with default parameters (Chen et al. 2018).

### 2.4 Plastome Assembly and Annotation

The plastome was assembled using GetOrganelle v1.7.5 (Jin et al. 2020) and annotated with CPGAVAS2 (Shi et al. 2019) and GeSeq (Tillich et al. 2017). We then manually adjusted the chloroplast genome data in Geneious Prime 2021.2.2 referring to the published plastome of *Stegnogramma sagittifolia* (NC035863; Wei et al. 2017). All tRNAs were verified using tRNAscan‐SE v2.0 (Chan and Lowe 2019). Circular genome maps were generated with OmicsSuite v1.3.9 (Miao et al. 2023). Four DNA regions (*mat*K, *rbc*L, *rps*16‐*mat*K, and *trn*L‐F) were extracted from the plastome and used for subsequent phylogenetic analysis.

### 2.5 Molecular Phylogenetic Analyses

Considering the close relationship between *Craspedosorus* and *Stegnogramma s.l*., two data matrices were generated: (1) plastome dataset consisting of 38 samples, and (2) a four plastid genes dataset consisting of 41 samples. Voucher information and GenBank accession numbers for the samples used in this study are listed in Tables 1 and 2. Matrices of the complete plastomes and each plastid region were aligned using Mafft v7.450 (Katoh and Standley 2013) and manually adjusted in BioEdit (Hall 1999). Based on the Akaike information criterion (AIC), ModelFinder (Kalyaanamoorthy et al. 2017) was used to select the best‐fitting models for both maximum likelihood (ML) and Bayesian analyses (BI). ML tree searches and ML bootstrapping were conducted using IQ‐tree v2.1.3 (Nguyen et al. 2015) with 5000 rapid bootstrap analyses followed by a search for the best‐scoring tree in a single run. BI was conducted using MrBayes v3.2.2 (Ronquist et al. 2012). Four Markov Chain Monte Carlo (MCMC) (one cold, three heated) were run, starting from a random tree. A total of 10,000,000 generations were executed, with sampling every 1000 generations. Convergence among generations was assessed using Tracer v1.7.1 (Rambaut et al. 2018) and the first 25% of samples from the cold chain were discarded as burn‐in. The remaining 7,500,000 trees were used to calculate a 50% majority‐rule consensus topology and posterior probability (PP) values. Equally weighted maximum parsimony (MP) analyses for each locus and the combined dataset were conducted in PAUP* v4.0b10 (Swofford 2002) using 1000 tree‐bisection reconnection (TBR) searches, with MAXTREES set to increase without limit. Gaps were coded as missing data. Parsimony jackknife (JK) analyses (Farris et al. 1996) were performed using PAUP*, with the removal probability set to approximately 37%, and “jac” resampling emulated. One thousand replicates were conducted, each with 10 TBR searches and a maximum of 100 trees held per search.

**TABLE 1:** List of plastomes used in this study.

Species	Voucher	Herbarium	Location	GenBank ID	Cities
*Abacopteris megacuspis* (Baker) Ching	Wei WQ224	KUN	Yunnan, China	MT130555	Du et al. (2021)
*Abacopteris nudata* (Roxb.) S.E. Fawc. & A.R. Sm.	Wei et al. FB374	KUN	Yunnan, China	MT130561	Du et al. (2021)
*Amauropelta beddomei* (Baker) Y.H. Chang	Cheng et al. FB475	KUN	Yunnan, China	MT130660	Du et al. (2021)
*Ampelopteris prolifera* (Retz.) Copel.	Cheng et al. FB040	KUN	Yunnan, China	MT130611	Du et al. (2021)
*Ampelopteris prolifera* (Retz.) Copel.	WR0326	PE	Yunnan, China	NC035835	Wei et al. (2017)
*Asplenium nidus* L.	Liu 2020	SYS	Cult. (SCBG)	NC045119	Cui et al. (2019)
*Christella acuminata* (Houtt.) Holttum	Unknown	Unknown	Unknown	NC070299	Unknown
*Christella appendiculata* (Wall. ex C. Presl) Holttum	WR035	PE	Yunnan, China	NC035842	Wei et al. (2017)
*Christella arida* (D. Don) Holttum	Unknown	Unknown	Unknown	NC070302	Unknown
*Christella dentata* (Forssk.) Brownsey & Jermy	JXJLS0001234	The Biological Herbarium of Jiangxi Provincial Management Bureau for Jiulian Mountain National Natural Reserve	Jiangxi, China	OM001014	Xu et al. (2023)
*Christella latipinna* (Benth.) H. Lév.	Unknown	Unknown	Unknown	NC070300	Unknown
*Christella parasitica* H. Lév.	Cheng et al. FB049	KUN	Yunnan, China	MT130695	Du et al. (2021)
*Christella parasitica* H. Lév.	Unknown	Unknown	Unknown	NC070301	Unknown
*Christella* sp.	Cheng et al. FB215	KUN	Yunnan, China	MT130565	Du et al. (2021)
*Coryphopteris japonica* (Baker) L.J. He & X.C. Zhang	Cheng et al. FB226	KUN	Yunnan, China	MT130553	Du et al. (2021)
*Craspedosorus sinensis* Ching & W.M. Chu	Zhou et al. YUS14506	PYU	Yunnan, China	C_AA133102	This study
*Cyclogramma auriculata* (J. Sm.) Ching	Wei WQ331	KUN	Yunnan, China	MT130552	Du et al. (2021)
*Cyclosorus interruptus* (Willd.) H. Itô	NIBRVP0000627878	National Institute of Biological Resources, Incheon, Korea	Jejudo Island, Korea	NC057240	Ramekar et al. (2020)
*Glaphyropteridopsis erubescens* (Wall. ex Hook.) Ching	Unknown	Unknown	Cult. (WBG)	MN623355	Liu et al. (2020)
*Glaphyropteridopsis erubescens* (Wall. ex Hook.) Ching	Wei WQ287	KUN	Yunnan, China	MT130562	Du et al. (2021)
*Grypothrix triphylla* (Sw.) S.E. Fawc. & A.R. Sm.	Unknown	Unknown	Cult. (FLBG)	MN623361	Liu et al. (2020)
*Macrothelypteris oligophlebia* (Baker) Ching	Lu Lu649	KUN	Zhejiang, China	MT130591	Du et al. (2021)
*Macrothelypteris torresiana* (Gaudich.) Ching	Liu. 201618	SYS	Cult. (SCBG)	MH500230	Zhou et al. (2018)
*Macrothelypteris torresiana* (Gaudich.) Ching	7471	PE	Guizhou, China	NC035858	Wei et al. (2017)
*Menisciopsis penangiana* (Hook.) S.E. Fawc. & A.R. Sm.	Cheng et al. FB227	KUN	Yunnan, China	MT130694	Du et al. (2021)
*Mesopteris tonkinensis* (C. Chr.) Ching	Liu 201617	SYS	Cult. (SCBG)	NC041428	Ding et al. (2018)
*Onoclea sensibilis* L.	WR0327	PE	Beijing, China	NC035860	Wei et al. (2017)
*Phegopteris decursive‐pinnata* (H.C. Hall) Fée	Unknown	Unknown	Cult. (FLBG)	MN623353	Liu et al. (2020)
*Phegopteris decursive‐pinnata* (H.C. Hall) Fée	Cheng et al. FB170	KUN	Yunnan, China	MT130548	Du et al. (2021)
*Pseudocyclosorus esquirolii* (C. Chr.) Ching	Cheng et al. FB527	KUN	Yunnan, China	MT130607	Du et al. (2021)
*Pseudophegopteris aurita* (Hook.) Ching	WR0326	PE	Jiangxi, China	KY427355	Wei et al. (2017)
*Pseudophegopteris pyrrhorhachis* (Kunze) Ching	Cheng et al. FB462	KUN	Yunnan, China	MT130575	Du et al. (2021)
*Pseudophegopteris yunkweiensis* (Ching) Ching	Cheng et al. FB127	KUN	Yunnan, China	MT130680	Du et al. (2021)
*Stegnogramma griffithii* (T. Moore) K. Iwats.	Cheng et al. FB137	KUN	Yunnan, China	MT130604	Du et al. (2021)
*Stegnogramma sagittifolia* (Ching) L.J. He & X.C. Zhang	7486	PE	Guizhou, China	NC035863	Wei et al. (2017)
*Trigonospora ciliata* (Wall. ex Benth.) Holttum	Wei et al. FB754	KUN	Hainan, China	MT130659	Du et al. (2021)
*Woodsia macrochlaena* Mett. ex Kuhn	Wu126	PE	Heilongjiang, China	NC035864	Wei et al. (2017)
*Woodwardia japonica* (L. f.) Sm.	NIBRVP0000524323	National Institute of Biological Resources, Incheon, Korea	Jejudo Island, Korea	NC050356	Ramekar et al. (2019)

**TABLE 2:** Vouchers and GenBank accession information for each species included in this study. A dash (—) indicates missing data.

Species	Voucher	Herbarium	Location	*trn*L‐F	*rbc*L	*rps*16*‐mat*K	*mat*K
*Abacopteris gymnopteridifrons* (Hayata) Ching	Kuo 857	TAIF	Taiwan, China	MN159524	MN159456	MN159488	MN159424
*Ampelopteris prolifera* (Retz.) Copel.	WR0326	PE	Yunnan, China	KY427329	KY427329	KY427329	KY427329
*Christella dentata* (Forssk.) Brownsey & Jermy	Kuo 960	TAIF	Taiwan, China	MN159523	MN159455	MN159487	MN159423
*Craspedosorus sinensis* Ching & W.M. Chu	Zhou et al. YUS14506	PYU	Yunnan, China	C_AA133102	C_AA133102	C_AA133102	C_AA133102
*Cyclogramma auriculata* (J. Sm.) Ching	Zhang 4455	PE	Yunnan, China	—	JN572338	—	—
*Goniopteris tetragona* (Sw.) C. Presl	Testo 791	VT	Heredia, Costa Rica	—	MN159459	MN159492	MN159428
*Leptogramma amabilis* Tagawa	Nakato 2705	TNS	Okinawa, Japan	MN159511	MN159445	MN159476	MN159411
*Leptogramma burksiorum* (J.E. Watkins & Farrar) Y.H. Chang & L.Y. Kuo	Eddie s.n.	Unknown	Alabama, America	MN159505	MN159439	MN159470	MN159405
*Leptogramma centrochinensis* Ching ex Y.X. Lin	Sanxia Exped. 1795	PE	Hubei, China	JN572303	JN572391	—	—
*Leptogramma chandrae* (Fraser‐Jenk.) Y.H. Chang & L.Y. Kuo	319749	TAIF	Meghalaya, India	MN159521	MN159454	MN159485	MN159421
*Leptogramma cyrtomioides* (C. Chr.) Y.H. Chang & L.Y. Kuo	Kuo 2126	TAIF	Sichuan, China	MN159517	MN159450	MN159481	MN159417
*Leptogramma dissitifolia* (Holttum) Y.H. Chang & L.Y. Kuo	Kuo 3566	TAIF	Mindano, Philippines	MN159506	MN159440	MN159471	MN159406
*Leptogramma himalaica* Ching	Liu 9476	TAIF	Yunnan, China	MN159520	MN159453	MN159484	MN159420
*Leptogramma intermedia* Ching ex Y.H. Chang & L.Y. Kuo	CYH20140714043	TAIF	Guizhou, China	MN159512	MN159446	MN159477	MN159412
*Leptogramma latipinna* (Ching ex Y.X. Lin) Y.H. Chang & L.Y. Kuo	Liu 9463B	TAIF	Yunnan, China	MN159518	MN159451	MN159482	MN159418
*Leptogramma leptogrammoides* (K. Iwats.) Y.H. Chang & L.Y. Kuo	Kuo 1426	TAIF	Yunnan, China	MN159519	MN159452	MN159483	MN159419
*Leptogramma mollissima* (Fisch. ex Kunze) Ching	Tagane & Tsujita TF592	TNS	Fukuoka, Japan	MN159510	MN159444	MN159475	MN159410
*Leptogramma mollissima* (Fisch. ex Kunze) Ching	Kuo 1009	TAIF	Tsukuba, Japan	MN159513	MN159447	MN159478	MN159413
*Leptogramma mollissima* (Fisch. ex Kunze) Ching	357631	TAIF	Tamil Nadu, India	MN159507	MN159441	MN159472	MN159407
*Leptogramma mollissima* (Fisch. ex Kunze) Ching	Kuo 110	TAI	Taiwan, China	MN159509	MN159443	MN159474	MN159409
*Leptogramma petiolata* Ching	000531335	BM	Sri Lanka	MN159508	MN159442	MN159473	MN159408
*Leptogramma pilosa* (M. Martens & Galeotti) Underw.	Pringle 2589	VT	Puebla, Mexico	MN159504	MN159438	—	—
*Leptogramma pilosa* var. *major* (E. Fourn.) Y.H. Chang & L.Y. Kuo	Testo 1070	VT	Oaxaca, Mexico	MN159503	MN159437	MN159469	MN159404
*Leptogramma pozoi* (Lag.) Heywood	LGQ 1095	MA	Coruna, Spain	MN159501	MN159435	MN159467	MN159402
*Leptogramma scallanii* (Christ) Ching	CYH20140712024	TAIF	Guizhou, China	MN159515	MN159448	MN159479	MN159415
*Leptogramma* sp.	Kuo 2238	TAIF	Sichuan, China	MN159516	MN159449	MN159480	MN159416
*Leptogramma totta* (Schltdl.) J. Sm.	00312042	P	La Convalescence, Comores	MN159514	—	—	MN159414
*Leptogramma tottoides* Hayata ex H. Itô	Kuo 3818	TAIF	Taiwan, China	MN159502	MN159436	MN159468	MN159403
*Macrothelypteris torresiana* (Gaudich.) Ching	7471	PE	Guizhou, China	KY427352	KY427352	KY427352	KY427352
*Meniscium reticulatum* (L.) Martyn	Testo 782	VT	Heredia, Costa Rica	MN159527	MN159458	MN159491	MN159427
*Metathelypteris uraiensis* (Rosenst.) Ching	Kuo 2347	TAIF	Taiwan, China	MN159525	MN159457	MN159489	MN159425
*Oreopteris quelpaertensis* Holub	Zhang 3583	PE	Jeju Island, Korea	—	JN572355	—	—
*Stegnogramma aspidioides* Blume	Wade 1902	TAIF	Java, Indonesia	MN159500	MN159434	MN159466	MN159401
*Stegnogramma dictyoclinoides* Ching	20140531‐1	TAIF	Taiwan, China	MN159499	MN159433	MN159465	MN159400
*Stegnogramma griffithii* (T.Moore) K. Iwats.	390559	TAIF	Meghalaya, India	MN159497	—	MN159463	MN159398
*Stegnogramma mingchegensis* (Ching) X.C. Zhang & L.J. He	Kuo 4240	TAIF	Fujian, China	MN159498	MN159432	MN159464	MN159399
*Stegnogramma sagittifolia* (Ching) L.J. He & X.C. Zhang	CYH20140714044	TAIF	Guizhou, China	MN159495	MN159430	MN159461	MN159396
*Stegnogramma wilfordii* (Hook.) Seriz.	20150329‐1	TAIF	Taiwan, China	MN159496	MN159431	MN159462	MN159397
*Steiropteris leprieurii* (Hook.) Pic. Serm.	Testo 1227	VT	Alajuela, Costa Rica	—	MN159460	MN159493	MN159429
*Thelypteris palustris* Schott	Larsson 16	UPS	Uppsala, Sweden	—	JF832085	—	JF832292
*Woodsia manchuriensis* Hook.	Zhang 2398	PE	Korea	KP226783	—	—	—

### 2.6 Ancestral State Reconstruction

For ancestral state reconstruction (ASR), we followed the same selection of samples and characters as Kuo et al. (2020). A total of 12 morphological characters, including 10 discrete and two continuous, were chosen. These characters are: (1) stipe indumentum, (2) rhizome habit, (3) venation, (4) basal pinnae, (5) lateral pinnules, (6) length of proximal pinnae, (7) maximum areole row number, (8) minimum areole row number, (9) maximum leaf dissection, (10) minimum leaf dissection, (11) maximum proportion of free vein pairs to the leaf margin, and (12) minimum proportion of free vein pairs to the leaf margin. Morphological characteristics of *Craspedosorus sinensis* were obtained from field observations and specimen studies. ASR analyses were performed in R v4.1.1 (R Development Core Team 2008) using phytools v0.7.80 (Revell 2012). Model selection was performed prior to stochastic character mapping. Three evolution models (Brownian Motion [BM], Ornstein–Uhlenbeck [OU], and Early Burst [EB]), for two continuous characters were fitted with “fitContinuous” command in the R package “geiger” (Pennell et al. 2014), and the best‐fitting model (Table S1) was selected by corrected Akaike information criterion (AICc; Burnham et al. 2002). Three different models (equal‐rates [ER], symmetric [SYM], and all‐rates‐different [ARD]) for ten discrete characters were fitted to the phylogenetic tree with “fitDiscrete” command in the R package “geiger” and best models (Table S2) were selected by AICc.

## 3 Result

### 3.1 Genome Size and Plastome Features of *Craspedosorus sinensis*

**FIGURE 2:**
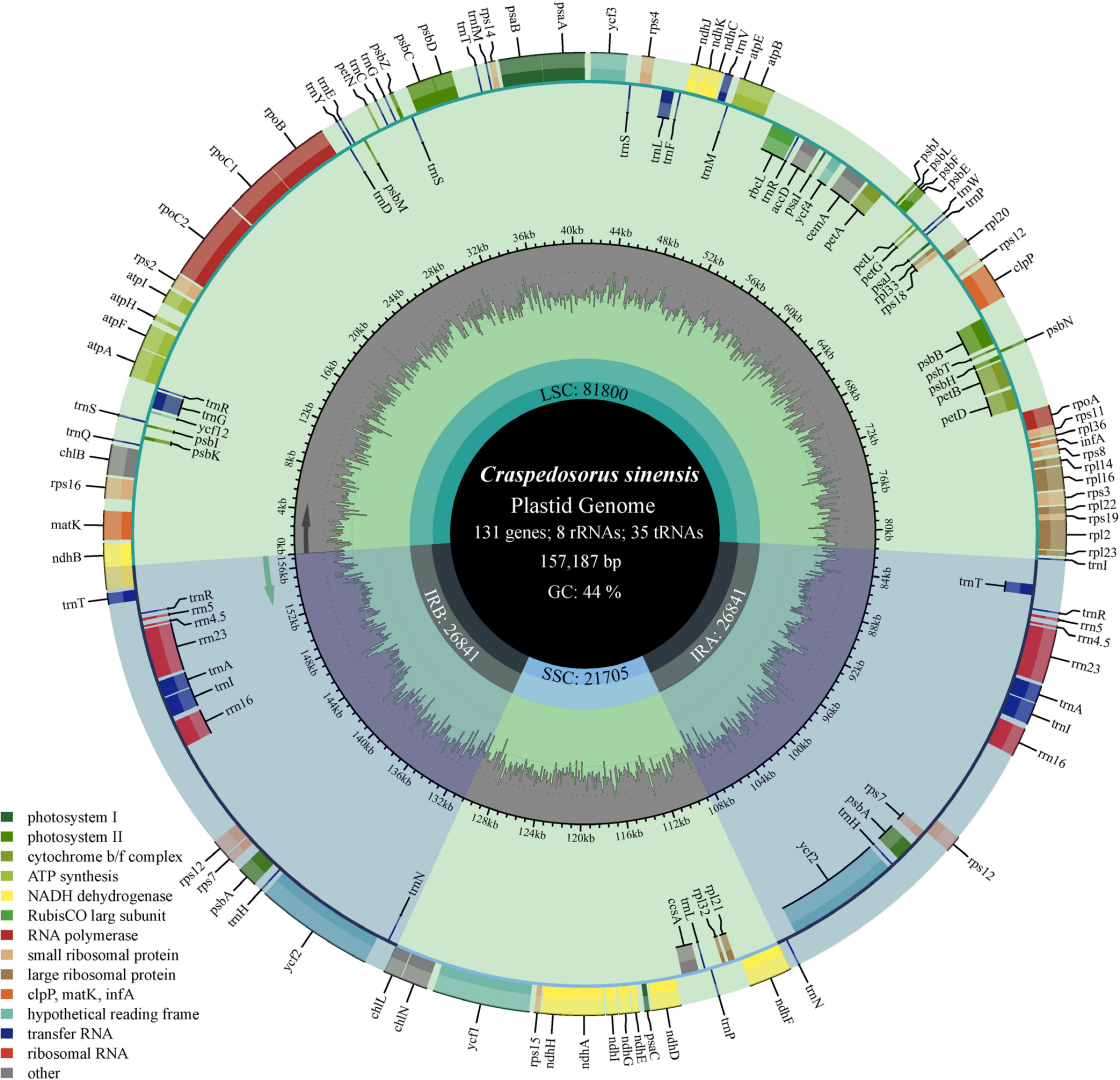
The plastome map of *Craspedosorus sinensis*. The dark gray track inside the map shows the GC content. Genes on the outside of the map are transcribed clockwise, and genes on the inside are transcribed counter clockwise. Genes belonging to different functional groups are shown in different colors; see the legend for groups.

Cytologically, the plant exhibited a DNA content of 5.12 Gb and was inferred to be diploid. The plastome of *Craspedosorus sinensis* sequenced here exhibits a typical quadripartite architecture, consisting of a large single copy (LSC: 81,800 bp), a small single copy (SSC: 21,705 bp), and two inverted repeats (IR: 26,841 bp) regions (Figure 2). The plastome contains 131 genes, including eight ribosomal RNA genes and 35 tRNA genes (Figure 2). The overall GC content of the plastome was 44.00% (Figure 2).

### 3.2 Phylogenetic Relationships of *Stegnogramma s.l*.

**FIGURE 3:**
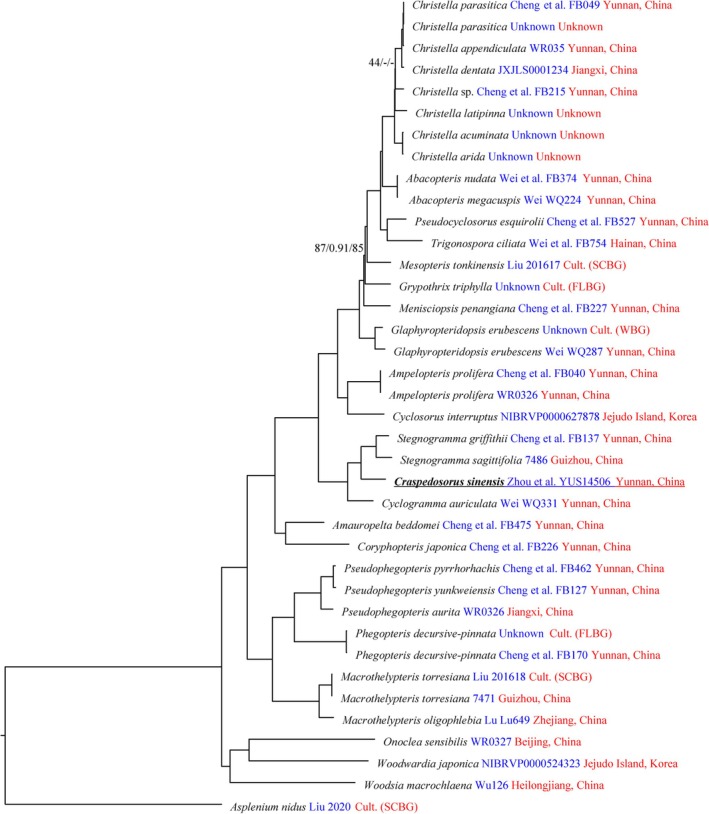
Maximum likelihood phylogeny of *Craspedosorus sinensis* based on the plastome dataset. Maximum likelihood bootstrap support (MLBS), maximum parsimony jackknife support (MPJK), and bayesian inference posterior probability (BIPP) are shown above the branches. Support values of 100 or 1.00 are not displayed.

**FIGURE 4:**
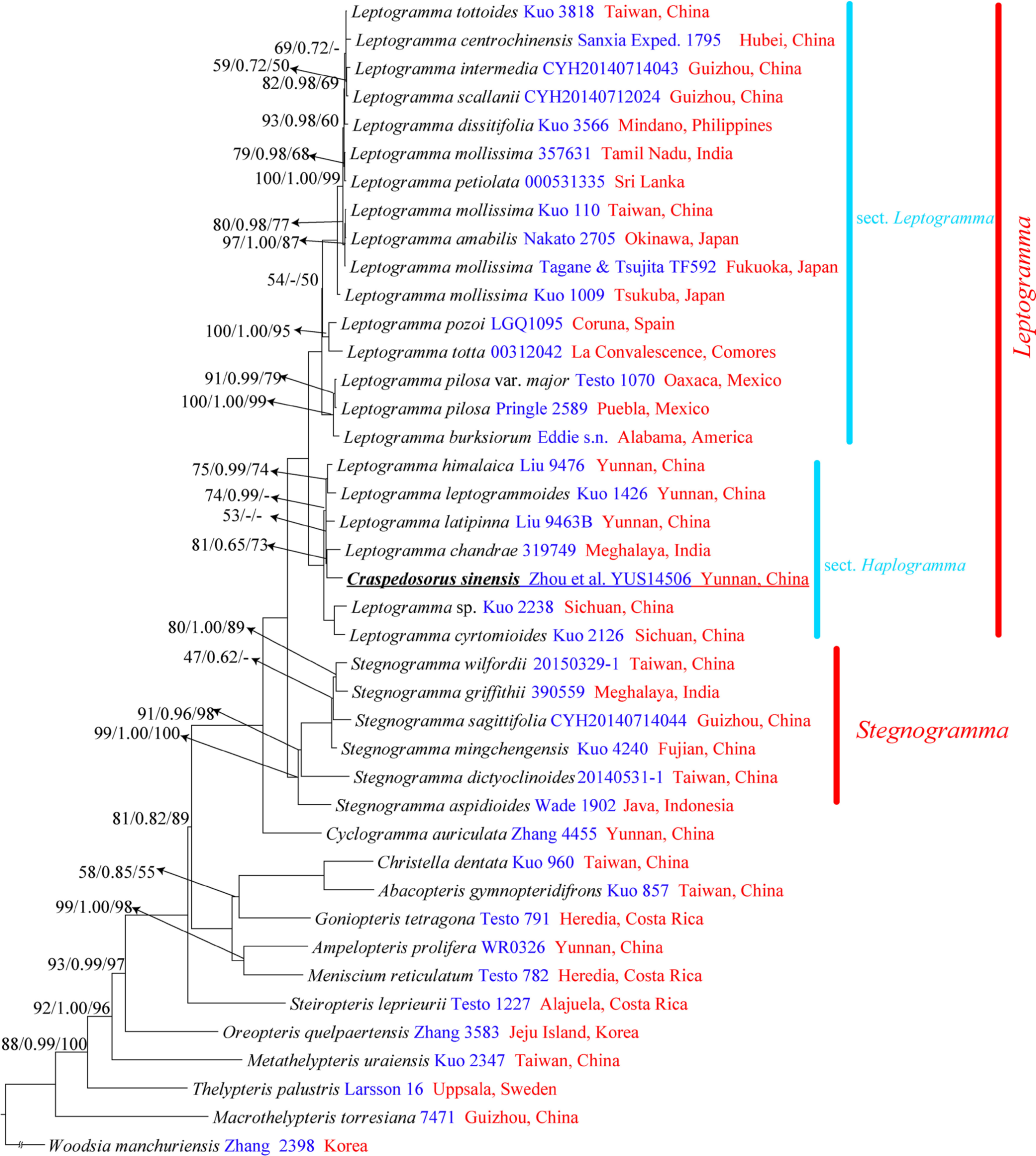
Maximum likelihood phylogeny of *Craspedosorus sinensis* based on the four plastid markers (*mat*K, *rbc*L, *rps*16‐*mat*K, and *trn*L‐F). Maximum likelihood bootstrap support (MLBS), maximum parsimony jackknife support (MPJK), and bayesian inference posterior probability (BIPP) are shown above the branches. Support values of 100 or 1.00 are not displayed.

MP, ML and BI analyses produced trees sharing the same general topology based on the same datasets (Figures 3 and 4). From our plastome and four plastid region datasets (Figures 3 and 4), *Stegnogramma s.l*. was consistently divided into two well‐supported clades. The tree topology derived from the plastome dataset fully supported the sister relationship between *Craspedosorus sinensis* and *Stegnogramma s.s*. (ML‐BS = 100, BI‐PP = 1.00, MP‐JK = 100; Figure 3). The inferred phylogenetic relationships based on the four plastid regions were highly consistent with the previous study by Kuo et al. (2020). The monophyly of *Stegnogramma s.l*. is also strongly supported (ML‐BS = 100; BI‐PP = 1.00; MP‐JK = 100) and is sister to *Cyclogramma* (Figure 4). In addition, *Stegnogramma s.l*. includes two well‐supported monophyletic clades (ML‐BS ≥ 99, BI‐PP = 1.00, MP‐JK = 100; Figure 4): *Stegnogramma s.s*. and *Leptogramma*. The *Leptogramma* lineage comprises two clades, *L*. sect. *Leptogramma* and *L*. sect. *Haplogramma*, which differ in the indumentum on the stipe. *C. sinensis* is well nested within the *Leptogramma* lineage, and *C. sinensis* is the sister group to *L. chandrae* with moderate support (ML‐BS ≥ 81, BI‐PP = 0.65, MP‐JK = 73; Figure 4). This represents the first molecular evidence for the position of *Craspedosorus*. However, the relationships among some species in *L*. sect. *Haplogramma* clade remain unclear, with some support values being moderate or even low (e.g., ML‐BS = 53, BI‐PP < 0.50, MP‐BS < 50; Figure 4).

### 3.3 Ancestral State of Morphology

**FIGURE 5:**
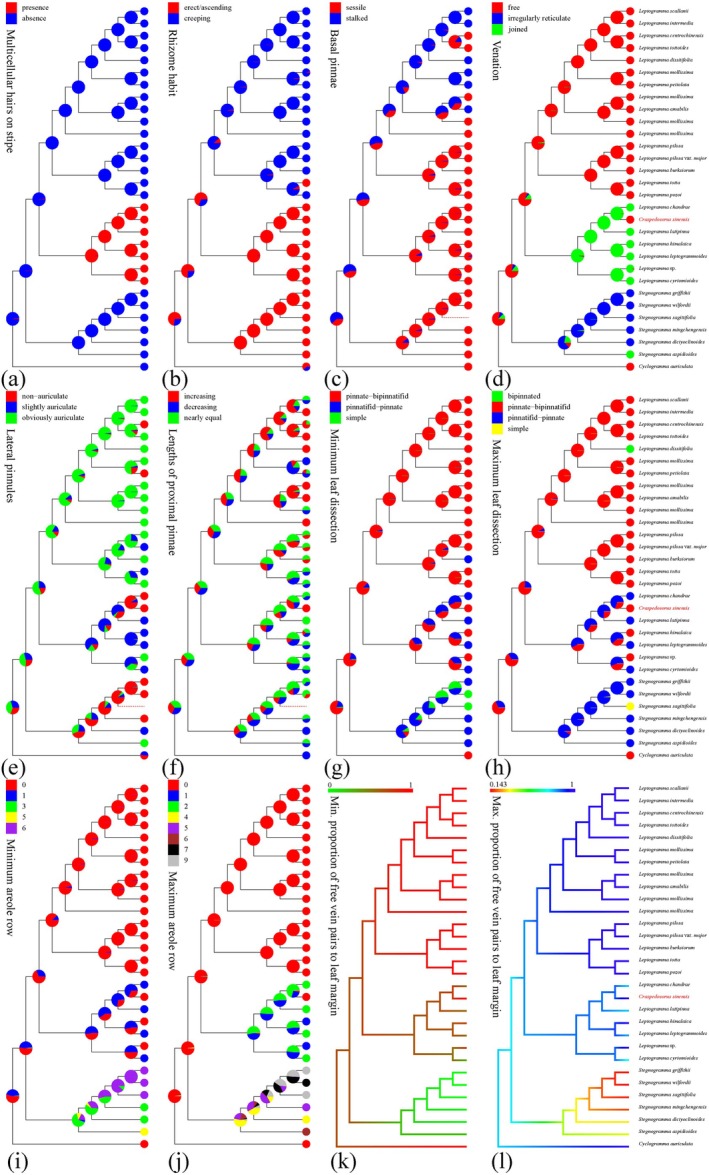
Ancestral character reconstruction of 12 morphological characters. (a) Stipe indumentum. (b) Rhizome habit. (c) Basal pinnae. (d) Venation. (e) Lateral pinnules. (f) Length of proximal pinnae. (g) Maximum leaf dissection. (h) Minimum leaf dissection. (i) Minimum areole row number. (j) Maximum areole row number. (k) Maximum proportion of free vein pairs to the leaf margin. (l) Minimum proportion of free vein pairs to the leaf margin. The pie charts on nodes summarize results of stochastic character mapping, and the color in each of the tip node shows the state.

We conducted a detailed morphological observation of *Craspedosorus sinensis* and performed ancestral state reconstruction. The results for 12 morphological characters are presented in Figure 5. The absence of multicellular hairs on the stipes represents the ancestral state in *Stegnogramma s.l*., whereas the presence of multicellular hairs on the stipes is a derived character in *L*. sect. *Haplogramma* (Figure 5a). Regarding the maximum areole rows, zero maximum areole rows represent the ancestral state in *Stegnogramma s.l*. while having more than one maximum areole row is an apomorphic character in *L*. sect. *Haplogramma* and *Stegnogramma* (Figure 5j). ASR analyses revealed that *C. sinensis* can be distinguished from closely related species of *L*. sect. *Haplogramma* by free venation, increasing lengths of proximal pinnae, and the absence of areole rows (Figure 5d,f,j).

## 4 Discussion

### 4.1 Phylogenetic Position of *Craspedosorus*

With the advancements in molecular technology, the publication of new taxon is now often accompanied by molecular data (e.g., Jiang et al. 2025; Zhao, Liang, et al. 2025; Zhao, Ma, et al. 2025; Zhou et al. 2025). *Craspedosorus*, a monotypic genus described by Ching (1978) based on morphological characters, was the only genus in China lacking molecular data due to limited material and field investigations. Although FRPS (Shing 1999) and FOC (Lin et al. 2013) recognized *Craspedosorus* as a distinct genus and considered it closely related to *Leptogramma*, the phylogenetic relationship between *Craspedosorus* and other genera of Thelypteridaceae remained unclear. Morphologically, *Craspedosorus* and *Leptogramma* are very similar in laminar outline, segment shape, venation, and sori. Recently, Kuo et al. (2020) proposed that *Craspedosorus* should be treated as a synonym of *Leptogramma* and further classified as a member of *L*. sect. *Haplogramma* based on morphological characteristics. Our inferred phylogeny also supports that *Craspedosorus* belongs to *Leptogramma* within the subclade *L. sect. Haplogramma* (Figure 4). The presence of multicellular hairs on the stipes further confirms that *C. sinensis* belongs to *L*. sect. *Haplogramma* (Figure 5a). *C. sinensis* is closely related to *L. chandrae* (Figure 4). However, *C. sinensis* differs from *L. chandrae* in several aspects: it is a larger plant, reaching nearly 100 cm (vs. up to 60 cm); it has free venation (vs. anastomosing venation with one or two areole rows); and its laminae are pinnate‐bipinnatifid (vs. pinnatifid‐pinnate). Furthermore, *C. sinensis* , which exhibits free venation, differs from other species of *L. sect. Haplogramma* in its venation pattern (free vs. anastomosing in *L. sect. Haplogramma*). Additional characteristics of *C. sinensis* are as follows: rhizomes erect; scales broad lanceolate, yellowish‐brown, with hairy margins; laminae broad lanceolate, pinnate‐bipinnatifid, not tapering at the bases; leaf margins membranous; basal pinnae sessile; and sori oblong, attached below ends of veinlets and close to margins (Ching 1978; Chu 2006; Figure 1). ASR analyses also indicate that *C. sinensis* is a relatively distinctive species both morphologically and phylogenetically within *Leptogramma* (Figure 5). Our results support treating *Craspedosorus* as a synonym of *Leptogramma*.

### 4.2 Morphological Evolution

The two genera, *Stegnogramma s.s.* and *Leptogramma*, can be distinguished by their venation patterns and the number of areole rows (Fawcett and Smith 2021; Figure 5). *Leptogramma* exhibits either free or anastomosing venation. Moreover, when venation is anastomosing in *Leptogramma*, the number of areole rows is limited to no more than two (Figure 5). Discrete characters such as rhizome habit, venation, leaf dissection, and the number of areole rows may have evolutionary significance related to environmental adaptation, geographical distribution, and other factors (Kuo et al. 2020). These discrete characters can effectively distinguish different clades from one other (Figure 5). The ancestral states of the remaining discrete characters in *Stegnogramma s.l*. appear to be uncertain, which also implies morphological diversity within the thelypterids (Figure 5).

Regarding continuous characters, the tips of the veinlets do not reach the leaf margin in *Craspedosorus sinensis*. However, this species, which has highly dissected fronds, exhibits an increased proportion of free vein pairs extending to the leaf margin. This pattern is consistent with the findings of Kuo et al. (2020), which suggest a correlation that it be correlated with latitude. For example, the frond of *C. sinensis* is pinnate‐bipinnatifid, with all free vein pairs reaching the leaf margin. According to collection records and field surveys, *C. sinensis* is distributed at high latitudes (Zhaotong, Yunnan, China, ca. N 28°21′ to N 28°40′).

### 4.3 The Protection of *Craspedosorus sinensis*

Although *Craspedosorus sinensis* was successfully identified using molecular evidence based on next‐generation sequencing technology, it has not been observed in the wild since 1973. Recently, we discovered a wild population in Yongshan County with ca. 15 adults. Based on the IUCN red list criterion (IUCN 2024), we classify it as Critically Endangered (CR). According to the Chinese government's policy document, the list of wild plants under special state protection includes a total of 11 families, 16 genera, and ca. 106 species of lycophytes and ferns. These species are either of significant application value or are relatively rare in the wild. With increased awareness of plant conservation in recent decades, more and more ferns have been effectively protected and rediscovered (e.g., rediscovery of *Cystoathyrium chinense* and *Angiopteris tonkinensis*) (Wei and Zhang 2014; Wang et al. 2020). This progress has also facilitated more in‐depth studies of many species (e.g., *Alsophila spinulosa*, *Ceratopteris richardii* , and *Isoetes taiwanensis*) (Huang et al. 2022; Marchant et al. 2022; Wickell et al. 2021). Therefore, we advocate that *C. sinensis* to be designated as a protected species in China in the future.

## Author Contributions

**Jing Zhao:** data curation (lead), formal analysis (lead), investigation (lead), methodology (lead), software (lead), validation (lead), visualization (lead), writing – original draft (lead), writing – review and editing (lead). **Jian‐Jun Yang:** data curation (lead), formal analysis (lead), investigation (lead), methodology (lead), software (lead), validation (lead), visualization (lead), writing – original draft (lead), writing – review and editing (lead). **Zhong‐Yang Li:** investigation (lead), resources (lead), software (equal), supervision (equal), validation (equal), writing – original draft (supporting), writing – review and editing (supporting). **Chuang‐Jie Huang:** investigation (supporting), resources (supporting). **Ting Chen:** investigation (supporting), resources (supporting). **Yong‐Li Zhao:** investigation (supporting), resources (supporting). **Zhao‐Rong He:** investigation (supporting), resources (supporting). **Chao‐Shan Gu:** funding acquisition (equal), investigation (lead), resources (lead), supervision (supporting), writing – original draft (lead), writing – review and editing (supporting). **Xin‐Mao Zhou:** conceptualization (lead), funding acquisition (equal), investigation (supporting), project administration (lead), visualization (supporting), writing – review and editing (lead).

## Funding

This work was financially supported by the Yunnan Fundamental Research Projects (Grant No. 202301BF070001‐016) and National Science & Technology Fundamental Resources Investigation Program of China (Grant No. 2022FY100201).

## Conflicts of Interest

The authors declare no conflicts of interest.

## Supporting information


**Table S1:** Model selection for ancestral state reconstruction based on two continuous characters. The bold font represents the best‐fit model.
**Table S2:** Model selection for ancestral state reconstruction based on ten discrete characters. The bold font represents the best‐fit model.

## Data Availability

The DNA sequences generated in this study have been deposited in the GenBase database of the China National Center for Bioinformation (CNCB). The accession numbers and the information on the voucher specimens are available in Tables 1 and 2.
